# Ecotones as Windows into Organismal-to-Biome Scale Responses across Neotropical Forests

**DOI:** 10.3390/plants13172396

**Published:** 2024-08-27

**Authors:** Perla Ortiz-Colin, Catherine M. Hulshof

**Affiliations:** Department of Biology, Virginia Commonwealth University, Richmond, VA 23284, USA; ortizcolinp@vcu.edu

**Keywords:** transition zone, edge, tropical dry forest, Cerrado, biome shift, upslope migration

## Abstract

Tropical forests are incredibly diverse in structure and function. Despite, or perhaps because of, this diversity, tropical biologists often conduct research exclusively in one or perhaps a few forest types. Rarely do we study the ecotone—the interstitial region between forest types. Ecotones are hyper-diverse, dynamic systems that control the flow of energy and organisms between adjacent ecosystems, with their locations determined by species’ physiological limits. In this review, we describe how studying ecotones can provide key indicators for monitoring the state of Neotropical forests from organisms to ecosystems. We first describe how ecotones have been studied in the past and summarize our current understanding of tropical ecotones. Next, we provide three example lines of research focusing on the ecological and evolutionary dynamics of the ecotone between tropical dry forests and desert; between tropical dry and rainforests; and between Cerrado and Atlantic rainforests, with the latter being a particularly well-studied ecotone. Lastly, we outline methods and tools for studying ecotones that combine remote sensing, new statistical techniques, and field-based forest dynamics plot data, among others, for understanding these important systems.

## 1. Introduction: What Is an Ecotone?

Ecotones can be considered natural laboratories for studying a range of evolutionary and ecological responses to contemporary global change [1]. They hold a special place in the history of ecology and evolution. Both Clements and Gleason studied the role of ecotones in the formation of plant communities at a time when ecology was nascent [2,3], and J. Huxley [4] demonstrated that hybridization and diversification can occur across ecotones. Ecotones control the flow of energy and organisms across landscapes and exist across a range of spatiotemporal scales [5]. The occurrence of an ecotone is generally determined by species’ physiological limits [6,7], often reflecting changes in environmental conditions such as edaphic or climatic factors. Because of their inherent spatiotemporal variability, studying and defining ecotones was historically challenging, requiring detailed and time-consuming field-based surveys [8,9]. Today, advances in statistical tools and technologies, like high-resolution satellite and drone-based imagery, present exciting opportunities to monitor ecotones in new ways and determine the underlying mechanisms driving their structure, function, and species composition. Arguably, the effects of global change drivers should be most evident and detected first in ecotones, making ecotones valuable early warning systems and climate change indicators [10,11].

Ecotone research has been relatively limited in tropical ecosystems, even though the tropics are experiencing some of the fastest rates of social, environmental, and climate change. Tropical forests are incredibly diverse, encompassing major climatic and geological gradients. Despite, or perhaps because of, this diversity, tropical biologists often ignore ecotones, choosing to study one or a few forest types. Some tropical ecotones, such as the savanna–forest and grassland–shrubland transitions, have been well-studied [12]. However, there are still major gaps in knowledge, for example, what drives the position and structure of the ecotone between dry and humid forests, two of the largest tropical biomes [13]. Because water availability is likely a key factor determining the transition between dry and humid forests, this and other ecotones across the Neotropics are especially vulnerable to future changes in precipitation regimes. In this review, we first briefly describe how ecotones have been studied worldwide at different spatial and temporal scales. We provide extra-tropical examples where monitoring ecotones has led to unique insight. We also emphasize techniques with obvious tropical applications, as well as outline challenges unique to monitoring ecotones in tropical ecosystems. Second, we review how ecotones have been studied in the tropics thus far, synthesizing our current level of understanding of these diverse and dynamic systems and highlighting areas where new tools and technologies could be applied. We focus on Neotropical examples, but also include examples from African and Australasian tropics when these seem most relevant to studying ecotones in tropical America. Lastly, we provide three example lines of research centered around the ecological and evolutionary dynamics of ecotones: between tropical dry forests and desert (Mexico); between tropical dry and rainforests (Costa Rica); and between Cerrado and Atlantic rainforests (Brazil), with the latter being a particularly well-studied ecotone. While we focus on terrestrial ecotones, equally important are aquatic ecotones (i.e., the deep chlorophyll layer) as well as ecotones between terrestrial and aquatic ecosystems (i.e., the salt marsh-upland forest ecotone) [7], especially as groundwater resources decrease and tidal regimes change. We hope this review serves as a guide for how future terrestrial tropical ecologists can expand their work to adjacent ecosystems and habitat types and the diverse ecotones between them.

## 2. Neotropical Ecotones: Our Current State of Understanding

Understanding the eco-evolutionary dynamics of ecotones is essential for determining the drivers of diversity and function of Neotropical forests, as well as predicting their responses to ongoing global changes. Over geological time scales and continental spatial scales, past responses of plants to climate change provide evidence that entire biomes, and thus the ecotones between them, can migrate, as seen in the expansion of South American Atlantic forests during the Pleistocene [14]. In other regions, vegetation belts exhibited altitudinal changes during the same period [15]. While it is easy to imagine an entire biome shifting in response to major climatic changes, biome shifts are the result of species-level responses [16]. Some species may decline or disappear, others may become more dominant, and yet others may diversify in response to novel selection pressures. Because biome shifts are evident through geological time and at continental scales, many models project biome shifts as a major outcome of climate change [6,17,18]. In many places this is already happening, as observed in North American grasslands [19], mangroves [20,21], West African Sahel [22], tropical moist forests and savannas [23], and elsewhere [24]. Despite much interest in biomes, especially as an ecological and biogeographical concept [25], the regions between them have received considerably less attention, a pervasive point we make here. Biome shifts imply that ecotones are experiencing equally significant shifts in location, properties, species composition, and population dynamics. In other words, a biome shift must be accompanied by a concomitant shift in the ecotones surrounding it. Recent evidence suggests that ecotones are expected to decline worldwide under all climate change scenarios, but model consensus remains low [26]. Model consensus is low likely because the factors that determine ecotone properties vary greatly. In temperate latitudes, the structure and position of treeline, a well-studied ecotone across Europe, is primarily controlled by thermal deficiency [27]. Conversely, tropical mountainous ecotones are often associated with a trade-wind inversion [28]. These examples and others highlight the importance of studying these ecologically significant intermediary systems.

Despite being understudied, a few generalities about ecotones have emerged. First, ecotones are areas of high species diversity. This is especially true across elevational gradients where high species richness often occurs between vegetation types. For example, bird diversity was greatest in ecotonal subalpine habitats in temperate Andes, Chile [29], and between tropical and temperate forests in the Sierra Madre Occidental, Mexico [30]. High species richness in ecotones has been attributed to area, temperature, energy, productivity, topography, and historical factors [31]. Additionally, ecotones tend to harbor unique species assemblages. Ecotones can be places where species characteristic of adjacent ecosystems co-occur [32] alongside species found nowhere else [33]. Importantly, ecotones are corridors for the movement and migration of organisms [34]. Nutrient cycling [35] and soil microbial communities [36] are also often distinct in ecotones compared to adjacent habitats. Differing nutrient dynamics and plant communities may point to underlying feedback processes that lead to the formation or maintenance of ecotones. Some studies report that ecotones exhibit increased productivity due to edge effects, though it is noted that natural ecotones are distinct from anthropogenic edges [37]. Although we focus on natural ecotones, we recognize that ecotones increasingly form as a result of fragmentation [38,39]. Thus, while some of these ecotone generalities apply to anthropogenic edges, edges have additional properties that make them unique cases.

In the Neotropics, most research on ecotones has been conducted on spatial scales ranging from the ecosystem to landscape and regional scales. Some studies, however, have focused on individual taxa [40] or organismal responses to the ecotone environment [41]. In addition, most of the research has focused on ecotones in the Amazon, particularly the forest–savanna ecotone. This body of research emphasizes complex interactions between vegetation types spanning contemporary responses to climate change, as well as long-term evolutionary processes shaping the biodiversity and composition of plant communities in the region. For example, several studies have used an evolutionary approach to analyze changes in temperature, precipitation patterns, and extreme weather events [12,16,42,43,44,45]. These studies provide evidence for the movement of the forest–savanna ecotone at the expense of forest during drier glacial periods. Other research in this region has focused on ecological drivers, dynamics, and contemporary impacts of climate change (i.e., [12,46,47,48,49,50]). This research has analyzed processes that maintain or influence ecotone characteristics including fire [51], herbivory [48], and soil properties [12]. At large scales, water availability determines the ecotone location, which is further mediated by local scale fire, herbivory, and soil properties. There is still debate, however, surrounding the importance of fire-grass feedback and feedback between shade and fire suppression, as well as how sensitive these ecotones are to contemporary climate change.

The dominance of Amazonian ecotone research can be attributed to several key factors. First, this region is a biodiversity hotspot and contains diverse ecotones, including tropical forest–savanna and tropical dry forest ecotones, rainforest–Andean cloud forest ecotones, and Cerrado–Atlantic forest ecotones [52]. These ecotones are ecologically significant as they exhibit distinct species assemblages and environmental gradients. The development of ecotones throughout Brazil is facilitated by the presence of soil and microclimate heterogeneity driven by topography, which enables different vegetation types to exist in proximity [23,33]. Second, Amazonian ecosystems are highly sensitive to climate change [53], both in geological and contemporary time scales, making them notable sites for studying the impacts of disturbances on ecotones. Since the last glacial period, savannas and dry forests have expanded, contracted, and expanded again, owing to major climatic shifts [42]. This cycle of biome expansion and contraction along ecotonal regions may indicate how present and future climate conditions will reshape ecosystems [46]. In response to contemporary drought, for example, rainforests may be replaced by dry forests or savannas, and cloud forests may move upslope [43,54]. Finally, ecotones in the Amazon (and elsewhere) play a crucial role in biodiversity conservation because they serve as corridors for species movement and genetic exchange as species reassortment occurs. Thus, it has been argued that conservation strategies in this region (and others) should include ecotones rather than emphasize biomes as homogenous [40,43,55]. The South Amazon Ecotones Ecological Corridor (Corredor Ecológico dos Ecótonos Sul-Amazônicos), proposed in 1997 [34], is one such example of conservation efforts specific to ecotones. The combination of exceptional biodiversity, a high concentration of ecotones across diverse biomes, and sensitivity to climate change has positioned the Amazon Basin as a hotspot for ecotone research.

Another hotspot of ecotone research in Neotropical forests occurs in the Andes. Here, research has similarly focused on reconstructing vegetation during past ice ages including forest–savanna dynamics and floristic composition in tropical montane forests and ecotones [15,46]. These studies highlight the probable uphill shift of modern montane forests, showcasing the sensitivity and ecological significance of tropical ecotones in the Americas. These areas are crucial reservoirs of biodiversity in the face of ongoing global changes [33,49]. Other research has shown that Cerrado and Chaco ecotones between Andean and Atlantic forests acted as refugia, impacting movement; gene flow; and, ultimately, the evolution of passerines [56]. Collectively, these authors have noted that species do not respond similarly. Therefore, species-level responses are an important component of understanding the complexities of species adaptations and the subsequent reassortment of entire communities and biomes along the ecotone interface.

The study of Neotropical ecotones in other regions is represented by either isolated case studies [49,57,58] or a focus on ecotones created by anthropogenic disturbances such as forest–pasture edges [55,59]. We argue that the unique properties of ecotones and their role in mediating biome shifts make them attractive systems for asking both fundamental and applied ecological and evolutionary questions, especially in the context of global change. In the next section, we highlight a few understudied areas.

First, very few studies have explored the role of plant functional traits in ecotones. One study describes tree wood density for species occurring in the forest–savanna ecotone of north central Roraima in northern Brazil [60,61]. Wood density influences growth, survival, and life history strategies, and it is important for calculating biomass and carbon estimates [62]. The Farias et al. [60,61] studies demonstrate an important feature of studying ecotones—to understand ecotone structure and dynamics, it is important to also evaluate adjacent habitats as reference points. In other words, it is often insufficient to study an ecotone in isolation from its adjacent habitats. A trait-based approach is also useful for understanding the mechanisms that maintain ecotone form and function. For example, across a New Zealand bog–forest ecotone, a trait-based approach revealed little differentiation across habitat types, suggesting that although species composition may vary between an ecotone and its adjacent habitats, plant form and function may not [63]. There is too little evidence to determine whether ecotones are functionally distinct or similar to adjacent habitats. Thus, trait-based approaches should focus on whether certain conditions lead to distinct (e.g., abrupt changes), gradual, or no measurable differences in ecotone properties relative to their adjacent habitats. Functional traits can also be used to determine how ecotone species cope with environmental gradients, varying competition, and climate change. For example, ecotone specialists may have a unique combination of traits that make them especially vulnerable to changes in environmental conditions—conditions found only within the ecotone. One approach for testing the degree to which ecotone species are vulnerable or resistant to change would be to compare traits of species which are characteristic of adjacent habitats, but which also co-occur in the ecotone.

Seedling dynamics offer another promising area of research for understanding ecotone dynamics. In the Neotropics, few studies have focused on ecotone seedling dynamics, though one study quantified seedling establishment, survival rates, and responses to environmental conditions at the treeline ecotone between upper montane tropical forests and alpine ecosystems [64]. Importantly, they showed a strong limitation for tree establishment in the páramo, maintaining treeline, but high rates of survival at the edge of the forest, which could lead to slow forest expansion as climate regimes shift. Studying seedling dynamics in other ecotones could serve as an early detection system for predicting future forest composition because seedlings are an important stage for niche differentiation [65]. An ecotone understory species composition that greatly differs from canopy or adult species composition, for example, may foreshadow major shifts in forest composition and function. The abiotic and biotic drivers of seedling recruitment have been used to predict treeline dynamics under climate change in temperate ecotones [66]. In the tropics, the vulnerability of seedlings to drought and the importance of the seedling stage for successful regeneration have been emphasized [67]. These studies demonstrate that understanding seedling responses to drought can inform forest regeneration and restoration. This may be especially important in ecotones. For example, in the forest–savanna ecotone of Ghana, tree seedling recruitment varied, and tree cover variation had species-specific effects on tree seedling recruitment, which was related to root storage [68]. Of course, studying the seedling layer requires considering other processes such as succession, herbivory, dispersal, and disturbances like fire [12].

Finally, there is a notable gap in research on species interactions within ecotones. This is true globally but could be an especially interesting line of research in tropical forests. The lack of research on ecological interactions within ecotones is surprising because ecotones are often contact zones between congeneric species [62], locations where species from distinct assemblages co-exist, and ecotones can even emerge as a direct result of species interactions [69]. As a result, ecotones are compelling models for studying competition, facilitation, and dispersal [70,71]. Studying competitive dynamics in ecotones could provide insight into species coexistence and species interactions at the edges of their ecological niches, especially under the added physiological stress caused by the distinct environmental conditions that characterize ecotones [71]. Other interactions such as pollination and seed dispersal could reveal biotic factors that favor or hinder plant distributions [41,72,73]. Williams et al. [72] demonstrated that local species richness of small, ground-dwelling mammals was explained by forest structure across a gradient from tropical rainforests to dry, sclerophyllous forests and woodlands. These mammals likely play a role in maintaining forest structure through dispersal. Trophic networks in ecotones may also reveal novel species interactions. For example, the higher species diversity of hummingbirds and plants in northwestern Mexico resulted in a more specialized network, but with lower nestedness and connectedness, indicating that ecotone species may be more vulnerable to disturbances [30]. In the next section, we outline key regions where addressing these and other questions could be especially fruitful.

## 3. Interesting Ecotones and Promising Research Questions

### 3.1. Mexico’s Pacific Dry Forests as a Biogeographical Bridge between Rainforests and Deserts

**Figure 1:**
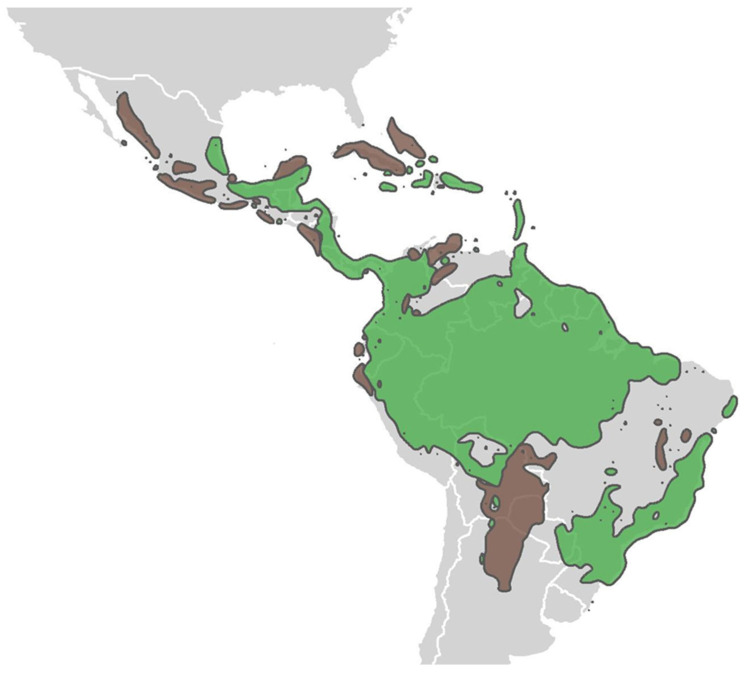
The distribution of neotropical dry (brown) and rainforests (green) suggests that ecotones should occur acrossthousands of kilometers across Mexico, Costa Rica, Colombia, Bolivia, Brazil, and smaller regions throughout. Ecoregion classifications were based on Olson et al. [80] for tropical and subtropical dry and moist broadleaf forests.

The antiquity of western Mexican dry forests (>20 million years before present) [74], and their unique position between wet forests and deserts, make them a prime location for studying the role of ecotones in biogeographical processes (Figure 1). This long history and ecological interface suggest dry forests may have served as a crucial biogeographical bridge for plant and animal migrations. For example, the fossil record hints at an evolutionary route from tropical wet forests, through dry forests, to North American deserts [75,76]. Studying Mexican dry forests could thus reveal biogeographical origins of floristic regions (e.g., [77,78]), vegetation shifts over geological timescales (e.g., [74]), adaptation mechanisms to drought and aridity [79], and potential migration pathways [76]. This information could be crucial for understanding the resilience of plant and animal communities experiencing contemporary climate change (like extreme heatwaves and freezes) in this region.

In addition, across vast stretches of the Americas, Africa, and southern Asia, a compelling pattern emerges: as rainfall decreases and becomes more seasonal, the prevalence of evergreen rainforests gives way to deciduous dry forests, woodlands, and ultimately deserts [81,82]. This climatic gradient suggests deciduousness evolved as a water-saving adaptation to seasonal drought [83]. Furthermore, leaf shedding during harsh conditions is considered a pre-adaptation to freezing temperatures, facilitating the migration of some plant lineages to colder climates (e.g., *Acer*, *Celtis*, *Magnolia*, *Prunus*, *Quercus*, *Rosa*, and *Salix*), possibly contributing to the development of temperate deciduous forests [84]. Others argue that these pre-adaptations played only a minor role in the evolution of temperate deciduous forests [85,86]. That freezing temperatures constrain the northern limits of dry forest distribution [87,88] also casts doubt on the extent to which tropical deciduousness facilitated northward expansion into colder regions. Even though temperate deciduousness and tropical deciduousness likely have quite distinct evolutionary histories and involve physiologically distinct processes, the emphasis on phenology from a temperate lens (the ‘temperate phenology paradigm’, [89]) limits our current understanding of tropical deciduousness. Thus, studying dry forests as an ecotone between rainforests and deserts could be used to understand evolutionary drivers of deciduousness and biogeographic patterns of phenology across large climatic gradients. Future research could emphasize phylogenetic relationships or divergence times between sister taxa and their leaf habits in tropical dry forests of northwestern Mexico and temperate or arid regions. Doing so may shed light on the timing and drivers of diversification, the selective pressures that shaped deciduousness, and the role deciduousness played in shaping biogeographical formations.

### 3.2. Cerrado and Atlantic Rainforest in Brazil: Linking Contemporary to Geological Change

Ecotonal regions between savannas and forests play a crucial role in biodiversity conservation. They span extensive areas at the intersections of significant biomes in South America, Africa, and Oceania, and they are home to a diverse array of species. One of the most emblematic ecotones in the Neotropics is the ecotone between Cerrado and Atlantic rainforests where vegetation elements of both systems co-occur. Predictive models showed that during the Last Glacial Maximum, the suitable areas of forests increased in this region due to a colder and more humid climate, whereas Middle Holocene forest cover likely diminished in area, before expanding once more as warmer and more humid conditions resumed [53]. Even though this is likely the most studied tropical ecotone, a few major gaps in knowledge persist. For example, the use of different bioindicators (isotopic, palynological, and phytolithic analysis) may help build more biologically realistic predictions for understanding expansion and contraction cycles of ecotones in response to climate change [53]. In addition, understanding local-scale ecotone processes is still limited, and questions regarding their properties remain unanswered [33]. There is still debate whether ecotones are distinct vegetation types or a transition between vegetation types. Souza et al. [33] found that ecotones between Cerrado and forests were distinct floristic units with a high number of unique species. Floristic and phylogenetic clustering further indicated that these ecotone environments are distinct vegetation types compared to core areas. This raises important questions about the characteristics and properties of ecotones and their ecological and evolutionary roles. Future work is needed to determine the conditions under which ecotones support habitat specialists rather than a mix of species from adjacent habitats.

### 3.3. Tropical Dry Forests and Rainforests: Ecological and Evolutionary Cousins

In the example of Mexico, we positioned the tropical dry forest biome as a large-scale ecotone between two other biomes, the Sonoran Desert and humid forests. In this final example, we describe the finer-scale ecotone that occurs at the boundary between tropical dry forests and rainforests. Ecotones between tropical dry forests and rainforests once occurred throughout the Pacific slopes of the Sierra Madre in Mexico and throughout the Cordillera Centroamericana [80] (see Figure 1). Today, most of this ecotone is badly degraded, making detecting this ecotone difficult. However, there are a few regions where this ecotone is largely intact—the Sierra de Manantlán Biosphere Reserve in central Mexico [90]; the southern slope of the Sierra Madre del Sur in Oaxaca [91]; and the Área de Conservación Guanacaste in northwestern Costa Rica [13,58] (Table 1). The Bolivian Chiquitano dry forest is also a notable ecotone, situated between the rainforests of the Amazon and the deciduous Gran Chaco [92,93]. The dry forest–rainforest ecotone is biologically important as a corridor for dry season migration [94,95,96] as well as for upslope migration due to climate change [97]. This movement of biota reiterates the importance of conserving ecotones as corridors for continued species reassortment. Because dry forest species are inherently drought tolerant [98,99], they may outcompete and eventually displace rainforest species, leading to an upslope expansion of the dry forest biome. However, this idea overlooks the possible built-in drought tolerance of rainforest species [100], the inability of wind-dispersed dry forest species to disperse upslope, against downslope tradewinds [101] or other interactions [70]. For example, if deciduousness evolved from rainforest species per theory [84], some rainforest species likely have a built-in capacity to withstand shifts in precipitation and temperature [100]. These possible responses make the ecotone between tropical dry forests and rainforests an especially interesting region for studying complex interactions between physiological tolerances and competition, especially as related to leaf habit. For example, dry forest species may outcompete rainforest species through stronger leaf shedding or more plastic phenological responses. Alternatively, rainforest species with deciduous strategies (facultative, brevi-deciduous, etc.) may adjust leaf shedding enough to keep pace with climate shifts. Mapping leaf habit in dry forest–rainforest ecotones and adjacent ecosystems can enhance our understanding of plant evolution and adaptation and inform strategies for conserving these biodiverse ecosystems.

## 4. How to Study Ecotones

### 4.1. Scale

**Figure 2:**
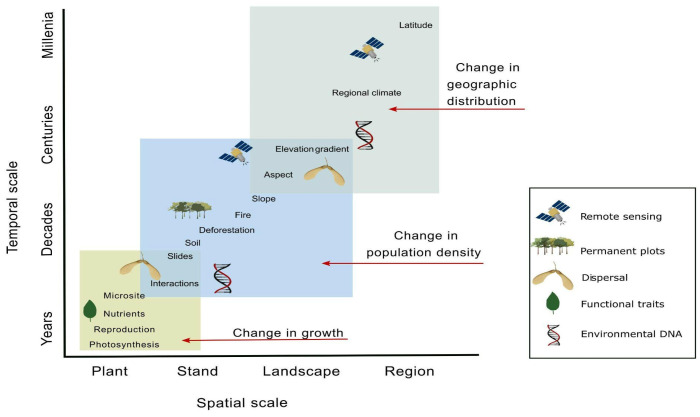
Conceptual diagram describing techniques for examining different questions across spatial and temporal scales in ecotones (modified from [10]).

Ecological systems exhibit variability across spatial, temporal, and biological scales, making scale a central problem in ecology [106,107]. Perhaps no other ecological system is as fundamentally tied to scale as ecotones [8]. Because ecotones can occur across a range of spatiotemporal scales, studying and comparing ecotones in a standardized way is challenging but presents an opportunity to design interdisciplinary approaches that could serve as a model for other systems (Figure 2). Simulation models are one such approach. Simulations connect life history traits, like dispersal, to broad-scale patterns of species abundances, diversity, and distributions across ecotones [108]. Other modeling approaches, like spatially explicit dynamical models, landscape spatial pattern analyses, and metapopulation models, can examine biotic and abiotic drivers that determine ecotone emergence, including how species, like ecosystem engineers, influence ecotone formation [69]. These approaches help to identify thresholds and scales at which different processes operate. Field-based approaches can also examine patterns and processes across scales and are particularly well suited for disentangling species–environment relationships. For example, field-based approaches can determine the interaction between landscape-scale geomorphological processes and local scale edaphic variation on species distributions [109], or the interaction between landscape-scale mesoclimate and local scale processes, like fire, on ecotone properties [57] (Table 2). Because life history strategies help organisms adapt to their environment based on their performance at different spatiotemporal scales, there are evolutionary implications of studying scale in ecological studies [107]. This makes ecotones model systems for the holistic study of organismal to ecosystem ecology and evolution.

### 4.2. Remote Sensing and Sensor Networks

Mapping species distributions and community composition within ecotones is a necessary first step for developing hypotheses about the underlying mechanisms and processes that drive ecotone structure and function. Doing so also enables interpretations of changes in spatial patterns and predictions of future shifts in response to ongoing climate and other change drivers. For this reason, remote sensing is a powerful tool for studying ecotones [116]. The application of remote sensing to the study of ecotones is usually combined with techniques for detecting areas of sharp transitions, or rates of change of some variable of interest (i.e., NDVI, the Normalized Difference Vegetation Index) across pixels. Studies also use remote sensing for land cover classification or clustering of vegetation types, typically identifying the ecotone as a distinct group. These techniques work well across abrupt changes in vegetation structure, for example across treeline [117] or across forest and savanna [118], but how well these methods work across forested areas, where changes are less evident, should be tested more broadly. Remote sensing imagery has been available for the past four decades, though the spatial and temporal resolution of earlier products may be too coarse for detecting ecotones. As a result, remote sensing is best used to monitor recent changes. In environments undergoing rapid land transformation, such as many tropical regions, remote sensing can be a useful tool for detecting these recent changes [119].

One study [58] leveraged seasonal phenological differences detected through remote sensing to monitor the ecotone for twenty years across a forested elevational gradient from tropical dry to rainforests in northwestern Costa Rica. In this study, the authors used spatial synchrony, or correlated temporal fluctuations, to classify forest types based on temporal phenological signatures. They showed the ecotone location is mediated by topography and precipitation seasonality; the ecotone is more dendritic along the tropical dry forest boundary; and while there was some evidence of ecotone upslope migration over the twenty-year period, inter-annual phenological responses were an important driver of ecotone dynamics. This study and others demonstrate the promise and challenges of using remote sensing to detect ecotones [120,121]. Specifically, spatial and temporal changes in ecotone properties detectable through satellite imagery reflect complex processes including phenological variation in addition to changes in (canopy) species composition.

Remote sensing techniques also tend to overlook fine-scale vegetation changes or individual tree responses, limiting their ability to assess local dynamics within ecotones [119]. However, the continued development of low-cost hyperspectral sensing and LiDAR-equipped drones are promising approaches for disentangling complex processes at finer spatial or temporal scales, thereby detecting subtler changes in ecotone forest structure and composition. Near-surface remote sensing and wireless sensor networks also enable the study of local to landscape-level processes, for example, using terrestrial backpack LiDAR [122]. Sensor networks could be deployed to monitor ecotone microclimates to address whether climate regimes are shifting faster in ecotones compared to their adjacent habitat types. Sensor networks distributed across different biomes, like the Terrestrial Ecosystem Research Network in Australia, encompassing tropical rainforests, wet and dry sclerophyllous forests, grasslands, and semi-arid habitats [123], are ideal locations for studying ecotones given the existing infrastructure, biophysical modeling, and ongoing monitoring in distinct habitat types. Together, these examples underscore the importance of ground-based research [57,124,125], even as technological advances enable studying processes across spatiotemporal scales.

### 4.3. Vegetation Dynamics Plot Networks

Despite the rise of technological advances in ecology [126], permanent plots remain necessary for understanding vegetation dynamics and for disentangling the mechanisms driving organismal to ecosystem-level changes (Figure 2). Long-term research enabled by permanent plot networks produces detailed records of tree growth, recruitment, and mortality, including the timing and cause of death of individual trees [125,127]. Long-term vegetation dynamics plot data would be especially useful for understanding ecotone dynamics. In particular, plot data could reveal interacting drivers of ecotone species composition changes in response to shifting climate regimes or identify plant populations that may be receding or expanding their distributions into neighboring habitats. A key benefit of permanent plot networks (like ForestGEO [128]) is the standardized methodology that facilitates comparisons across spatial and temporal scales. For example, Sterck et al. [93] used a network of 220 one-hectare plots across Bolivia encompassing a large precipitation gradient. While not explicitly focused on ecotones, this gradient includes dry forests at the transition between Amazonian evergreen rainforests in the north and the Gran Chaco thorn scrub in the south. This network, and others like it, enables research focusing on ecotone dynamics.

Permanent plots are also useful for understanding how abiotic factors determine the establishment and maintenance of ecotones. Ecotone theory largely predicts that the high species turnover found in ecotones across environmental gradients is associated with changes in abiotic variables. Thus, discrete ecotones along environmental gradients should be associated with pronounced environmental discontinuities (e.g., soil parent materials, abrupt topographic changes) or associated with a history of contrasting disturbances. To test this theory, quantitative studies of species composition should be replicated across spatial scales, as achieved by Martin et al. [57] in the Cordillera Central, Dominican Republic. Here, the authors used vegetation sampling at the plot scale and gradient analyses at the landscape scale to determine if vegetation change was gradual or discrete across an elevational gradient. They reported that fire at local scales drove the discreteness of the ecotone, but that mesoclimate drove fire occurrence patterns.

Forest dynamics plots can also be used to address questions about the underlying conditions driving tree recruitment and mortality, as well as how these are shaped by biotic factors in addition to abiotic ones. In subtropical and tropical forests of Yunnan, Southwest China, Lin and Cao [129] observed that abiotic conditions at forest edges notably affect the distribution and behavior of soil seed banks and understory vegetation, highlighting how ecotone characteristics can determine species dynamics. Furthermore, biotic factors such as interspecies competition among seedlings may outweigh the influence of abiotic factors like temperature within ecotones [130]. Biotic factors like soil microbial communities may also play an important role in ecotone dynamics and climate change responses. Networks like GlobalFungi [131] provide useful resources for the inclusion of soil microbes into forest dynamics monitoring and other long-term plot networks established within and across ecotones.

### 4.4. Dispersal

In addition to biotic interactions like competition, seed dispersal likely contributes to high species turnover and shapes community structure within ecotones. Yu et al. [132] reported that rodent-mediated seed dispersal significantly influenced tree species composition and recruitment dynamics in a pine–oak ecotone between subtropical and warm-temperate regions in central China. Plant dispersal is a key component of establishment and, in the context of climate change, an important component of species migration [133]. Thus, studying dispersal is an important component of understanding potential future shifts in ecotones. Agent-based simulation models, for example, can demonstrate how different types of plant dispersal (i.e., wind, long distance, gravity, or others) lead to different spatial patterns of species moving into new habitats (or not) and, at larger scales, of ecotones advancing across an environmental gradient [134]. Wind dispersal is one such example that will likely lead to unique spatial patterns of species movement [135]. This is especially true where lowland dry forests intersect with upland rainforests along the Pacific slopes of Mexico and Central America. Here, the upslope migration of wind-dispersed dry forest species may be constrained by wind patterns. The dominant wind direction is downslope [136], against the presumed direction of upslope climate tracking. Detailed mechanistic wind dispersal models [137] could be used to study complex interactions between atmospheric conditions and plant traits (like dispersal mode) that influence the spatial pattern of seed dispersal across ecotones.

Finally, in an eco-evolutionary context, demographic population dynamics, gene flow, and local adaptation are inherently tied to dispersal [138,139] and should also be considered when studying climate-induced ecotonal species movement or movement of the entire ecotone (see Table 2). Stochastic patch occupancy models could determine the interplay between ecological and evolutionary dynamics as related to dispersal [140]. Moreover, it has recently been suggested that the evolution of dispersal may be determined by landscape structure and spatial variation; these two factors appear to have more influence over dispersal than emigration strategies [141]. This finding points to the importance of landscape pattern on dispersal in different types of ecotones, as well as to the dispersal constraint that species may face when moving from fractal landscapes (gradual ecotones) to mosaic landscapes (transition zones with sharp-edged patches). Ecotones are thus excellent places for studying metapopulation dynamics and complex eco-evolutionary feedback. Ecotones could also serve as systems for bridging the gap between theoretical predictions on the evolution of dispersal and empirical data (as outlined by [142,143]).

### 4.5. Physiological Responses to Temperature and Drought

Because species’ physiological limits determine ecotone locations [46], studying plant ecophysiology in the ecotone is key for understanding responses to ongoing and future climate change. Drought is one of the most severe stressors for species and ecosystems worldwide, requiring rapid assessment of drought tolerance [144]. An improved mechanistic understanding of drought responses will enhance the accuracy of models predicting drought-induced forest mortality. Such knowledge is also valuable for land management, guiding choices about which tree species to plant and optimal tree population density [145]. This is especially relevant in ecotones where many plant species may exist at their hydraulic safety margins, enabling tests of predictions from future drought scenarios.

Leaf water potential at turgor loss point (TLP), or wilting, effectively predicts drought responses across biomes [144]. This makes TLP a candidate trait for measuring across ecotone species. However, assessments of plant ecophysiology (or any other trait) of ecotone species necessitate the measurement of the same traits in adjacent vegetation types to contextualize species performance across a range of environments. Moreover, monitoring the impact of extreme events, such as drought or fire, can reveal species-specific responses. For example, different species exhibited different physiological responses to an extreme drought in northern Arizona’s forest–woodland ecotone [146]. Similar approaches in the tropics could reveal long-lasting changes in species dominance within ecotones, as well as how these changes may cascade to impact adjacent habitats and other trophic interactions.

Further, the role that phenotypic plasticity and trait variation play in plant responses to temperature and drought stress can easily be studied in ecotones, providing additional insights into the resilience and adaptability of plant species. Plants may exhibit a greater range of intra- and inter-specific trait variation in ecotones due to more variable conditions, though this has not been well studied [147]. Quantifying relationships between physiological trait variation and environmental variation within ecotones and their adjacent habitats will further advance our understanding of species distributions and species ranges across environmental gradients and the filters driving community assembly.

While in situ measurement of physiological traits in ecotones and their adjacent habitats is an important component of testing climate-change-related hypotheses, a comprehensive, interdisciplinary approach is more likely to reveal complex interactions between climate, plant physiological responses, and habitat dynamics. Greenhouse experiments, for example, could better reveal the drivers of species survival or mortality. Apgaua et al. [148] exposed seedlings of three *Eucalyptus* species from different habitats (wet forest, savanna, and forest–savanna ecotone) to drought, elevated temperature, and a combination of elevated temperature and CO_2_ concentration. In drought conditions, savanna seedlings survived longer than their forest and ecotone counterparts. The ecotone species had a heightened sensitivity to elevated temperatures compared to forest and savanna species. To cope with this sensitivity, the ecotone species increased their stomatal conductance to cool down leaves. Given that these ecotone species are already close to their moisture threshold, rising temperatures could elevate their water usage, potentially accelerating mortality during severe drought [148]. Experiments like this one, in combination with the measurement of physiological traits, could be used to predict plant responses to climate change.

### 4.6. Environmental DNA Metabarcoding

Environmental DNA (eDNA) metabarcoding is a cutting-edge technique for studying landscape genetics because it is a non-invasive and efficient method for monitoring biodiversity (including vascular plants communities) through the sampling of soil, water, or air [149,150]. Because this technique promises to improve the scale of data collection while reducing effort and investment of resources and time, it is especially useful for studying communities of organisms that are difficult to tell apart using conventional methods, such as fungi or bacteria in soil samples. For instance, metabarcoding was used to characterize ectomycorrhizal fungal communities associated with different tree hosts in a boreal–temperate ecotone [151]. By analyzing DNA sequences from fungal-colonized root tips, they were able to identify changes in fungal community composition in response to warming and precipitation experiments.

EDNA metabarcoding could be valuable for tracking genetic flow across ecotones and neighboring habitats, providing insights into how species adapt and potentially diverge while still exchanging genes. In addition, ecotones are often hybrid zones where pairs of closely related species co-occur, providing opportunities for gene flow and speciation [152] (Table 2). If ecotones contain unique and endemic species and alleles, it could support the idea that these areas are significant in the process of speciation. Under this hypothesis, ecotonal regions would likely host a majority of newly evolved species (neoendemics) that have not yet expanded their geographical ranges [1]. Nuclear eDNA methods may permit estimates of population allele frequencies and abundances [153] within ecotones, revealing the evolutionary processes that have helped to make ecotones biologically distinct systems of high importance to biodiversity maintenance [33].

## 5. Conclusions

Neotropical ecotones, such as those between tropical dry and rainforests experiencing ongoing climate and other anthropogenic disturbances, warrant urgent study. These ecotones act as biodiversity reservoirs, concentrating a diverse biota from adjacent ecosystems within a relatively small area. Given their role in speciation, ecotones might be considered the true cradles of biodiversity [1]. However, as biomes shift due to climate change, ecotones will experience similar or greater changes in species composition and other properties. Their inherent variability makes predicting the outcomes of climate change drivers on ecotones challenging with traditional models or tools. Therefore, interdisciplinary approaches are necessary for studying ecotones. Remote sensing, simulations, and other modeling approaches should be integrated with field-based research, ecophysiological measurements, and genetic data. Furthermore, studying ecotones alongside their adjoining ecosystems is essential for understanding ecotone formation and function, as well as for determining the extent to which ecotones can serve as early indicators of the impacts of global change. Ecotones are model systems for addressing ecological and evolutionary questions across hierarchical spatial, temporal, and biological scales. We hope this review provides a roadmap for such research to integrate long-term monitoring and prediction with a focus on biodiversity and speciation and ecotone responses to global change.

## Figures and Tables

**Table 1:** Candidate regions for studying tropical dry forest–rainforest ecotones.

Country	Region	Relevant Studies
Mexico	Sierra de Manantlán Biosphere Reserve, central Mexico; the southern slope of the Sierra Madre del Sur in Oaxaca	[90,91]
Costa Rica	Área de Conservación Guanacaste, northwestern Costa Rica	[13,58,97]
Colombia	La Sierra Nevada de Santa Marta; Cauca-Patía Basin; Magdalena River Basin	[102,103,104]
Bolivia	Santa Cruz, Bolivia	[93,105]
Brazil	Caatinga-Cerrado-Atlantic ranforest ecotones, northeastern Brazil	[23,33]

**Table 2:** Scale-related relevant topics for studying ecotones.

Scale	Focus	Example Studies and Key Findings
Stand	Impact of microhabitat on species composition	The transition zone between tropical moist and dry forests significantly influenced epiphyte composition (vascular and non-vascular), but microsite conditions affected only non-vascular epiphytes [110].
	Plant trait variation	Functional trait diversity showed a mosaicity pattern in the ecotone, indicating that the spatial heterogeneity of functional traits within transition zones played a crucial role in defining the ecological dynamics of bog-forest ecotones [63].
Landscape	Soil carbon dynamics	Microbial activity and nutrient availability were key drivers of soil carbon dynamics in a forest–savanna ecotone in Ghana [111].
	Edge effects on plant recruitment	The negative edge effect on seedling recruitment in a forest and grassland ecotone was attributed to reduced seed availability, unfavorable post-dispersal conditions for germination, and seedling establishment [112].
	Environmental filters	Plant community trait values shifted in response to soil and light variation. Low soil nutrients and water in the coniferous zone were major constraints for most lowland rainforest species with acquisitive traits [113].
Region		
	Disturbance	Tropical forests and savannas worldwide may represent alternative stable states, with their resilience universally varying based on precipitation. Tree cover responded non-linearly to changes in precipitation [114].
	Speciation	Quaternary climate variations influenced population divergence including genetic differentiation due to forest contraction and biome separation between Amazonia and Atlantic Forests. These climate-driven divergences, occurring in recent times, also explained speciation and evolutionary radiation over longer timescales [115].

## Data Availability

No new data were generated by this work.
